# Reviewer acknowledgement

**DOI:** 10.1186/s12993-016-0094-z

**Published:** 2016-03-11

**Authors:** Vivienne A. Russell

**Affiliations:** University of Cape Town, Rondebosch, Cape Town, 7700 South Africa

## Contributing reviewers

The editors of *Behavioral and Brain Functions* would like to thank all our reviewers who have contributed to the journal in Volume 11 (2015).

**Athena Akrami**

USA

**Branko Aleksic**

Japan

**Silvia Alemany**

Spain

**Mareike Altgassen**

Germany

**B. Bai**

China

**E. Baldi**

Italy

**Pauline Baniqued**

USA

**Simon Baron-Cohen**

UK

**G. Bjorklund**

Norway

**Fausto Bogazzi**

Italy

**Arun Bokde**

Ireland

**Cesar Borlongan**

USA

**Balint Botz**

Hungary

**Marco Bove**

Italy

**Carmela Bravaccio**

Italy

**G. Britton**

Panama

**Natascia Brondino**

Italy

**Heather K. Caldwell**

USA

**Patrick Callahan**

USA

**Alessandro Castorina**

Italy

**Miguel Cervantes**

Mexico

**Chia-Yen Chen**

USA

**Jg Chen**

China

**Joungil Choi**

USA

**Ori Cohen**

USA

**Veronique Coizet**

France

**Maria Collu**

Italy

**M. Teresa Colomina**

Spain

**Erika Comasco**

Sweden

**James Connor**

USA

**Heather Cordell**

UK

**Marilyn Cornelis**

USA

**Jacqueline Crawley**

USA

**Anita Cservenka**

USA

**Elena Daprati**

Italy

**Tania Das Banerjee**

USA

**Minet De Wied**

The Netherlands

**Birgit Derntl**

Germany

**Darragh Devine**

USA

**Linda Douw**

The Netherlands

**Diana Dow-Edwards**

USA

**Benjamin Dunn**

Norway

**Ulrich Ettinger**

Germany

**Julia Fedotova**

Russian Federation

**Michaela Filiou**

Germany

**Laura Fusar Poli**

Italy

**Abby Fyer**

USA

**Patrick Gajewski**

Germany

**Daniela Galashan**

Germany

**Pablinny Galdino**

Brazil

**S. Gandevia**

Australia

**Carles Gaston-Massuet**

UK

**Ivan Gentile**

Italy

**I. Griskova-Bulanova**

USA

**Yvonne Groen**

The Netherlands

**Jiang Gui**

USA

**Aymeric Guillot**

France

**Matthias Hammerschmidt**

Germany

**Bernhard Hommel**

The Netherlands

**Iqbal Hossain**

USA

**Fleur Margaret Howells**

South Africa

**Lucian Hritcu**

Romania

**Yong Hu**

Hong Kong

**Ming-Chao Huang**

USA

**Peter Hubbard**

Portugal

**David John Hume**

South Africa

**Edvin Ingberg**

Sweden

**Yossef Itzhak**

USA

**Yasuhiko Izumi**

Japan

**Michelle Jacobs**

USA

**Vivek Jeevakumar**

USA

**Matthew Johnson**

USA

**Miriam Johnson**

UK

**Jukka Jolkkonen**

Finland

**Am Kaindl**

Germany

**Wendy Kalberg**

USA

**Wendy Kates**

USA

**S. Kawato**

Japan

**Mathilakath Keralapurath**

USA

**Janet Kern**

USA

**Hubert Kerschbaum**

Austria

**Sahib Khalsa**

USA

**Peter R. Killeen**

USA

**Hs Kim**

Republic of Korea

**Mitchiteru Kitazaki**

Japan

**Andrea Kóbor**

Hungary

**Eva Kocovska**

UK

**Michael Kolch**

Germany

**E. Kubinyi**

USA

**Autumn Kujawa**

USA

**Kate Langley**

UK

**David Lawrence**

USA

**Tatia M. C. Lee**

China

**Bombi Lee**

Republic of Korea

**Boreom Lee**

Republic of Korea

**Elisabeth Leehr**

Germany

**V. Leoni**

Italy

**Joanna Lewin-Kowalik**

Poland

**Jarrod Lewis-Peacock**

USA

**Shuxin Li**

USA

**Zhaomin Li**

USA

**Bjorn Liaset**

Norway

**S. Lin**

USA

**Yuan Shao Lin**

China

**Jun Liu**

China

**Sara Lopez-Martin**

Spain

**Elizabeth Losin**

USA

**Astri J. Lundervold**

Norway

**Jinglei Lv**

China

**Birajara Machado**

Brazil

**Jane Maddock**

UK

**Petra Majdak**

USA

**Kailash Manda**

Japan

**A. Martin**

Spain

**Salvador Martínez**

Spain

**Ivo Marx**

Germany

**Laura Marzetti**

Italy

**Maurizio Mattia**

Italy

**Natasha Maurmann**

Brazil

**James Mckenna**

USA

**Katja Menzler**

Germany

**S. C. Meredith**

USA

**Frank Middleton**

USA

**Gabriela Morali**

Mexico

**Katsumi Nakajima**

Japan

**Hiroki Nakata**

Japan

**Shaimaa Nasr**

Egypt

**Eric Nelson**

USA

**Kiyotaka Nemoto**

Japan

**Ujjwal Neogi**

Sweden

**Gerald Nestadt**

USA

**Caroline Nievergelt**

USA

**Michael Noll-Hussong**

Germany

**Micheal Nunez**

USA

**John O'Toole**

Spain

**Robert Oades**

Germany

**Ennio Ongini**

Italy

**Esther Opmeer**

The Netherlands

**Rajendra Parajuli**

Canada

**Robert Paul**

USA

**Fernando Pena-Ortega**

Mexico

**Zw Peng**

China

**Golshani Peyman**

USA

**Bettina Pfleiderer**

Germany

**S. Pittaccio**

Italy

**Sanjay Prakash**

India

**Victor Raggio**

Uruguay

**Rajamannar Ramasubbu**

Canada

**Janez Ravnik**

Slovenia

**Marta Re**

Italy

**Greg Reynolds**

USA

**Pamela Rist**

USA

**Jill Roberts**

USA

**Tania L. Roth**

USA

**Julia Rucklidge**

New Zealand

**Philipp Ruhnau**

Italy

**Jong Hoon Ryu**

Republic of Korea

**Renee Sadowski**

USA

**Melita Salkovic-Petrisic**

Croatia

**Jack Samuels**

USA

**C. Scassellati**

Italy

**Maria Scherma**

Italy

**Steven Schwartz**

USA

**Sarojini Sengupta**

Canada

**Manu Sharma**

Germany

**Christopher Shaw**

Canada

**I.-Hsuan Shen**

USA

**Jack Shepard**

USA

**Kyungsoon Shin**

Republic of Korea

**Hee-Sup Shin**

Republic of Korea

**Alain Simard**

Canada

**Elizabeth Simpson**

USA

**Ionara Siqueira**

Brazil

**Neil Smalheiser**

USA

**E. Sobarzo-Sanchez**

Spain

**Lin Sørensen**

Norway

**Viola Stoermer**

USA

**Drozdstoy Stoyanov**

Bulgaria

**Irina Stoyanova**

The Netherlands

**Jakob O. Ström**

Sweden

**Matthew Sutherland**

USA

**Zsuzsa Szombathyne Meszaros**

USA

**Samuel Sam Wah Tay**

Singapore

**Mahendra Thakur**

India

**Matthias Treder**

UK

**George Tsirakis**

Greece

**Cortney Ann Turner**

USA

**Kristin Uhler**

USA

**Thomas Vaissiere**

USA

**E. Van Duinkerken**

The Netherlands

**Marieke Van Vugt**

The Netherlands

**Alberto Velez-Van-Meerbeke**

Colombia

**L. Vendruscolo**

Brazil

**Ganesan Venkatasubramanian**

India

**Rajkumar Verma**

USA

**Rishma Vidyasagar**

Australia

**Sophia Vinogradov**

USA

**Shivakumar Viswanathan**

Germany

**Styliani Vlachou**

Ireland

**Joanne Voisey**

Australia

**Sallyann Wakeford**

UK

**Stephan Waldert**

UK

**Lihong Wang**

China

**J. Wang**

China

**Bo Wang**

China

**Zi-Jun Wang**

USA

**E. Wascher**

Germany

**Tony Wilson**

USA

**Ryan Wong**

USA

**Jinglong Wu**

Japan

**Semon Wu**

USA

**Z. Xi**

USA

**Miyoung Yang**

Republic of Korea

**Takao Yasuhara**

Japan

**Lee Yong-Seok**

USA

**Shan Ping Yu**

USA

**Ying Zang**

China

**Jacqueline N. Zanoni**

Brazil

**Dóra Zelena**

Hungary

**Ruixin Zhang**

USA

**Yu Zhang**

USA

**Jiqiang Zhang**

China

**Yanli Zhang-James**

USA

**June Zhou**

USA

